# Interdisciplinary Education: The Advanced Science Research Center

**DOI:** 10.1016/j.isci.2020.100822

**Published:** 2020-01-22

**Authors:** Joshua C. Brumberg, Annette C. Gray

**Figure:**
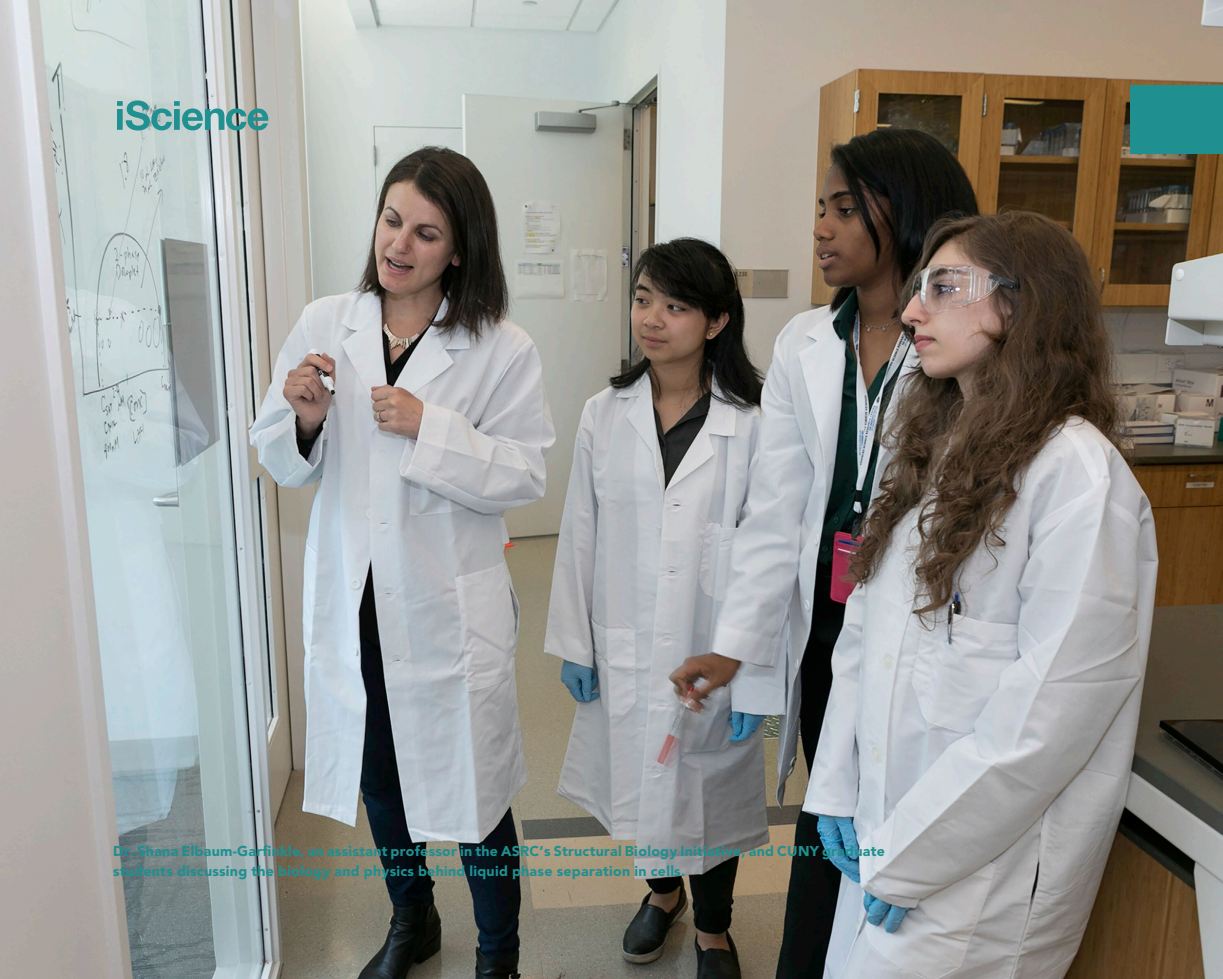
Dr. Shana Elbaum-Garfinkle, an assistant professor in the ASRC's Structural Biology Initiative, and CUNY graduate students discussing the biology and physics behind liquid phase separation in cells.

The Advanced Science Research Center at the Graduate Center of The City University of New York (CUNY ASRC), established in 2014, has two primary goals. The first is to provide CUNY scientists with state-of-the-art core facilities that enable them to create novel materials and devices as well as perform experiments with the intellectual and technical support of experts. The second is to promote interdisciplinary interactions across a multitude of disciplines. To achieve these goals, the center has established 15 core facilities ranging from functional magnetic resonance imaging to nanofabrication and supports scientists from five diverse fields: nanoscience, photonics, structural biology, neuroscience, and environmental sciences.

Below, Joshua Brumberg, Dean of the Sciences at The Graduate Center, and Annette “Nina” Gray, Associate Dean of the Sciences at The Graduate Center and Executive Director at CUNY ASRC, discuss both the benefits and the challenges of running an interdisciplinary research institution.

## Proximity: Recruiting the Right People

Once you have the right people in place, you need them to interact, and proximity matters.

Josh: The key factor has been recruiting scientists who are willing to think outside of the box and willing to work with individuals outside of their own communities. Once you have the right people in place, you need them to interact, and proximity matters. A recent example we have of this working is a collaboration between our Mass Spectrometry Core (part of the Structural Biology Initiative) and our Live Cell Imaging Core (part of the Neuroscience Initiative) to create a novel resource for the New York City scientific community (https://asrc.gc.cuny.edu/neuroscience/facilities/maldi-imaging-joint-facility/)

Nina: We are also mindful that, independent of exploring interdisciplinary research collaborations, time can be a major challenge for faculty. This is especially the case for junior faculty who are still establishing themselves and seeking tenure. Senior faculty need to be sensitive to their junior colleagues' need to balance individual research programs with collaborative efforts, both in terms of being good collaborators and in terms of evaluating their progress as mentors and supervisors.

## Language: Providing a Platform for Cross-disciplinary Communication

Josh: The biggest issue I've observed here has been people not wanting to look foolish by asking basic questions, but I think we have activities that help curtail this fear of embarrassment. For example, when I ask someone what PDE stands for—physicists and engineers say partial differential equations, whereas biologists say phosphodiesterase. To help bridge this communication gap we have weekly “Science Cafés” hosted by one of our five initiatives. In these cafés, someone gets up and gives a short talk about what they do in a way that everyone can understand. We are taking a similar approach with graduate students, where we are increasingly asking them to give short talks (1 slide, 3 min) to groups of other students not in their fields as a way of getting them to fine-tune their elevator talks to a general audience.

Nina: These “Science Cafés” promote interactions among scientists at all levels. In addition to learning more about the research being conducted throughout the center, it's been a great opportunity for new members to meet everyone and immediately become integrated into the community. The building also features informal meeting spaces on all the floors so that impromptu “water cooler” conversations can easily happen. A prerequisite for successful interdisciplinary collaboration is to get people speaking the same scientific language with the same meaning.

A prerequisite for successful interdisciplinary collaboration is to get people speaking the same scientific language with the same meaning.

Additionally, our scientists, particularly graduate students, are frequently engaged in public outreach where they talk about their research to vastly different audiences. This helps them learn how to approach the challenge of working across scientific disciplines, now and in their future careers.

**Figure:**
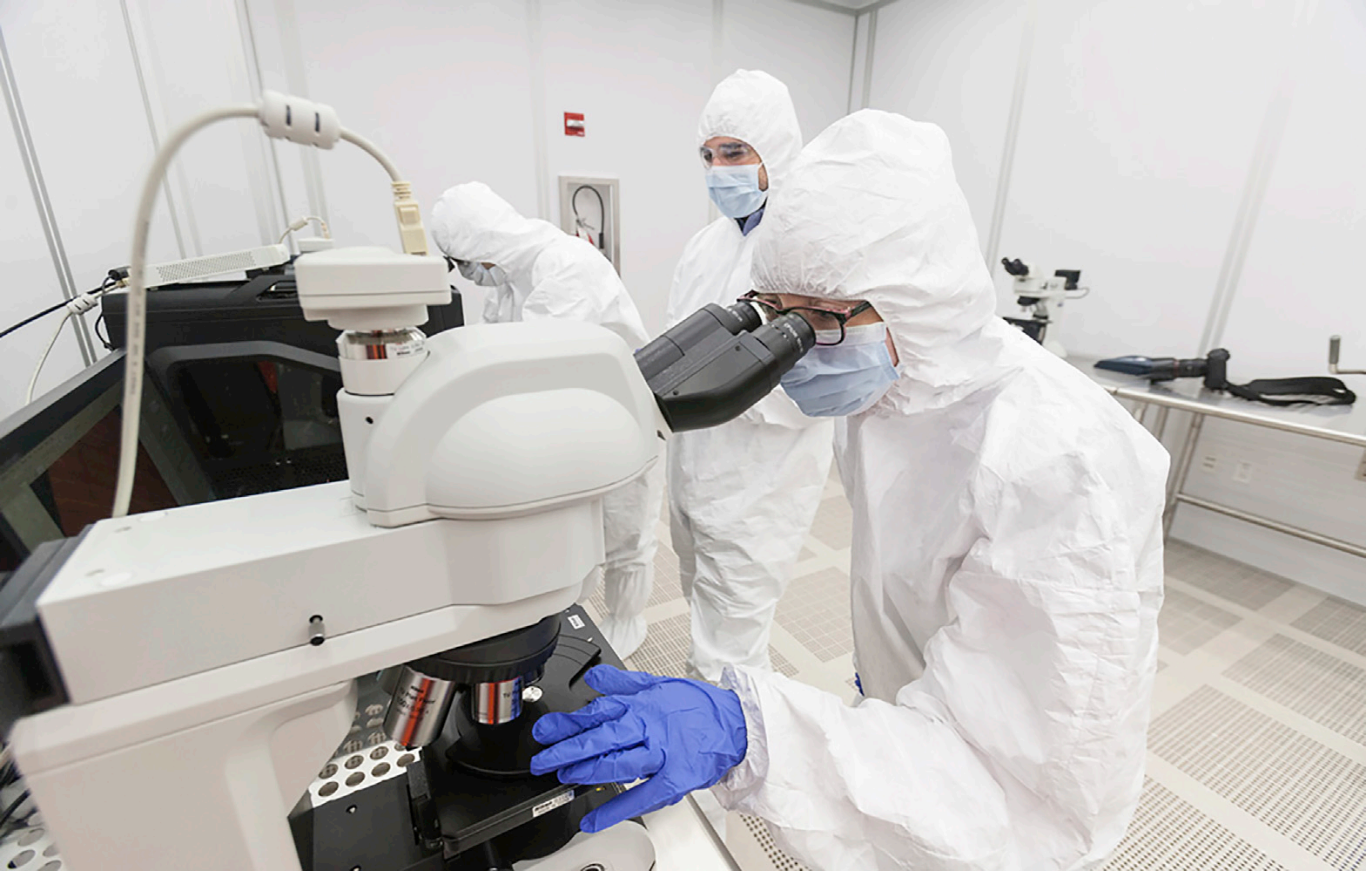
Researchers in the ASRC's Nanofabrication Facility, one of the center's 15 core research facilities.

## Research Methods: The Value of Mentorship

Nina: To be successful in collaborations, learning new techniques is often necessary—or at least learning enough to understand the pros and cons and how to interpret and evaluate data from collaborators. Having a cohort of experienced core facility directors, who are research faculty, has been a big help in making sure that students receive ample training doing experiments and analyzing data so that they feel confident enough to do so independently.

Good communication from the outset is crucial, and we try to build a community here that values open dialogue and is inclusive of everyone's voice, regardless of what stage of their career they are at.

Josh: I think for PhD students, the only real concern is that they get spread too thin—Jack of all trades, master of none-type phenomenon. We emphasize that students get proper mentorship—and that might mean two mentors across different disciplines—which provides students with strong foundations upon which interdisciplinary interactions can be built.

## Governance: Good Project Management Requires Good Communication

Josh: To successfully manage a project, I think it is important to not be too formal. Since each project is unique, we often recommend spending some time at the outset of any project or collaboration discussing individual roles and expectations. This allows researchers to define time commitments and to sketch out a feasible timeline for the project that considers outside factors as well.

As far as securing funding goes, one tool we have deployed is seed funding that prioritizes interdisciplinary interactions among CUNY scientists. We have provided over $700,000 in support of CUNY scientists working on these interdisciplinary projects. Providing funds for early-stage projects has allowed CUNY scientists to gather pilot data to successfully apply for larger external grants and thereby continue and expand their research programs.

We are increasingly training our students and faculty to be able to speak about their science with the public.

Nina: Good communication from the outset is crucial, and we try to build a community here that values open dialog and is inclusive of everyone's voice, regardless of what stage of their career they are at. Apart from making sure students feel empowered enough to speak up, we also have a lot of students with co-mentors from different initiatives or departments, and they are inherently part of collaborative interdisciplinary research teams and gain first-hand experience sitting at the table to guide and manage complex projects.

## Publication: Reaching Broad Audiences

Nina: This is a significant challenge—it can be hard to reach an audience within a single discipline given the volume of research being published now. Social media and other online platforms are the best tools we have right now, and I think we are just beginning to tap into them. For example, I'd like to see us create more short video abstracts for broad audiences. Eventually, a collection of these can also become a resource for future interdisciplinary training.

Josh: We are increasingly training our students and faculty to be able to speak about their science with the public. We have hosted workshops with comics to teach improvisation skills, workshops on writing op-ed pieces and using social media, as well as creating a series of talks explicitly marketed to the general public (the most recent example: https://www.gc.cuny.edu/All-GC-Events/Calendar/Detail?id=52701)

**Figure:**
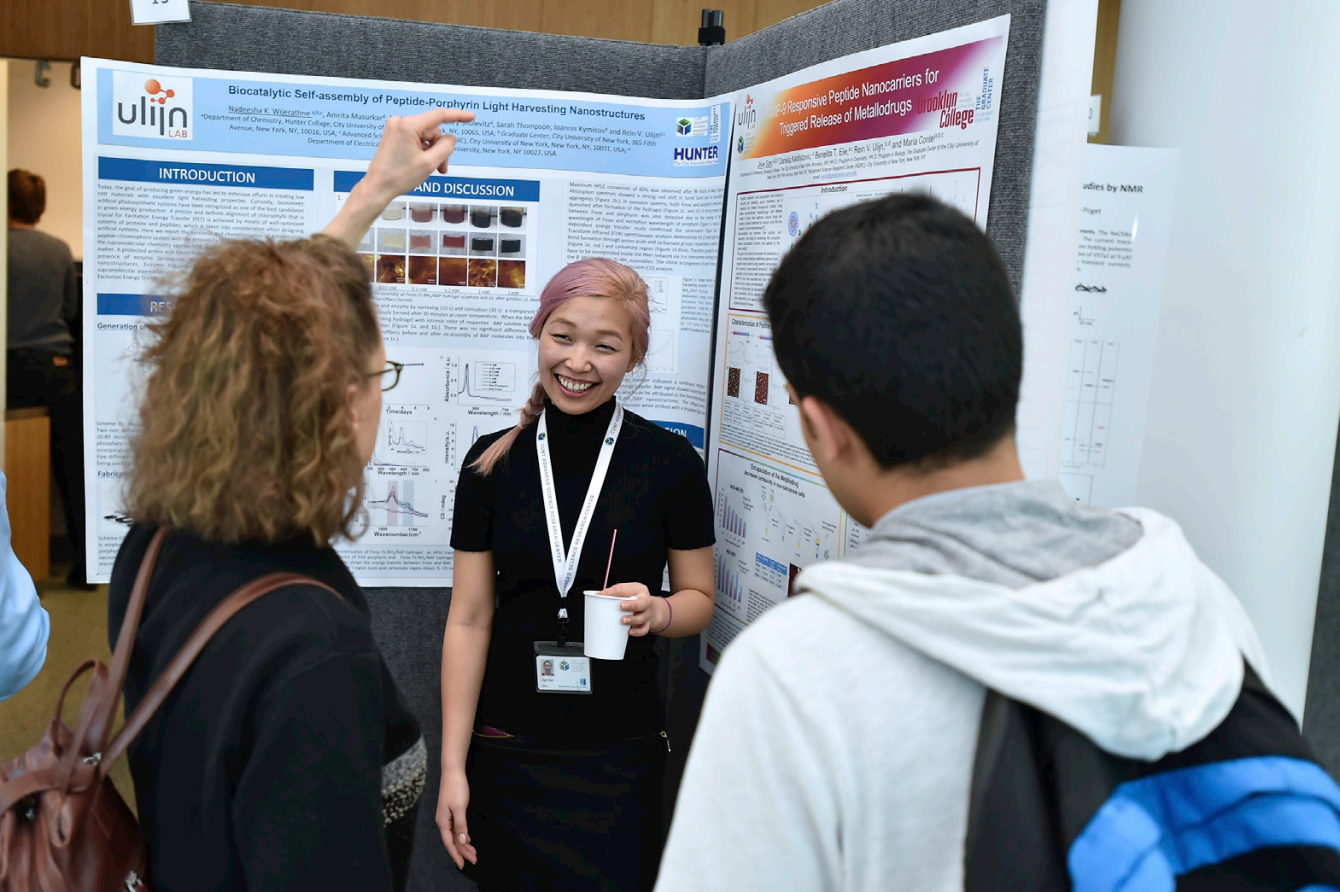
Dr. Jiye Son, a recent graduate of The Graduate Center's Program in Chemistry, presents her research during an open house at the ASRC.

## Conclusion: Final Thoughts

Josh: I believe that for science to continue to advance we will have to bring together researchers from different communities and that will be the focus of the next 5 years at the CUNY ASRC. Although science continues to progress within individual disciplines, the real excitement is when scientists from different backgrounds bring their shared expertise to solve important societal problems. For example, sustainability requires environmental scientists to assess what is happening within our environments, which is aided by collaborating with nanoscientists and photonics specialists who can help engineer those devices. In turn, the impact of the environment on living organisms requires neuroscientists and structural biologists to evaluate the biological ramifications. By housing these varied disciplines under one roof, we aim to address these big issues in truly interdisciplinary ways.

Nina: We are just getting started exploring the possibilities of interdisciplinary research at the ASRC and CUNY, and we need to continue to make sure intellectual curiosity and important questions drive the conversations and research. This spring, in a symposium, we will explore research directions, opportunities, and challenges we currently face and lessons we can learn from other organizations. I think the conversations we will have will stimulate a new wave of activity and creativity, which will keep us very busy.

